# Clinical and radiological evaluation of inverse impaction of supernumerary teeth

**DOI:** 10.4317/medoral.18877

**Published:** 2013-05-31

**Authors:** Elif B. Tuna, Esma Kurklu, Koray Gencay, Gulsum Ak

**Affiliations:** 1 DDS, PhD. Associate Professor, Department of Pediatric Dentistry, Faculty of Dentistry, Istanbul University, Istanbul, Turkey; 2 DDS, PhD. Research assistant Dr., Department of Oral Surgery, Faculty of Dentistry, Istanbul University, Istanbul, Turkey; 3 DDS, PhD. Professor, Department of Pediatric Dentistry, Faculty of Dentistry, Istanbul University, Istanbul, Turkey; 4 DDS, PhD. Professor, Department of Oral Surgery, Faculty of Dentistry, Istanbul University, Istanbul, Turkey

## Abstract

Objective: To describe the clinical and radiological features of children with inverted supernumerary teeth. Study Design: Thirty eight patients with inverted supernumerary teeth (ST) were enrolled in this descriptive and restrospective study. Data from patient records including age, gender, status of dentition, number of ST, number of ST in inverted position, coexistence of ST in inverted and normal direction of eruption, location, orientation, morphology, clinical complications, management and radiography were assessed during 3-years period. Results: Thirty eight patients with a mean age of 9.10±1.97 years (range:6-13) and a strong male preponderance of 3.7:1 (male:30, female:8) had a total of 69 ST, of which 41 were in inverted position. Thirty five patients had one (92.1%) inverted tooth, whereas 3 patients had two inverted teeth per case (7.9%). All cases were located in the maxilla. Midline was the most frequent site for the single inverted supernumerary tooth in 18 (47.4%) patients, followed equally by the right and left premaxillary region in 10 patients each (26.3%). Regarding morphology, 30 patients had conical (78.9%) and 8 (21.1%) had incisiform ST. No tuberculate shaped ST was detected. There was no statistically significant difference between number of inverted teeth and delayed tooth eruption, diastema, local malocclusion, palatinal swelling (p>0.05). There was no statistically significant difference between complications and age (p>0.05). Surgical removal at the time of diagnosis with subsequent follow-up during completion of permanent dentition was the treatment approach in all cases. Conclusions: Thorough clinical examination followed by a comprehensive radiographic screening is the crucial determinant of an accurate diagnosis of an impacted ST. Early diagnosis and timely management are key factors to prevent or minimize the complications, which may influence function and esthetics of the teeth and even psychological condition of the growing child.

** Key words:**Supernumerary tooth, impaction, inverted.

## Introduction

ST are defined as any tooth or odontogenic structure that is formed from a tooth germ in excess of the usual number for any given region of the dental arches and may present in both the permanent and primary dentitions (1). These are developmental abnormalities occasionally encountered in the clinical practices of pediatric dentistry and oral surgery. Prevalence of occurrence has been reported to range between 0.15-3.9% for permanent and 0.2-0.8% for primary dentition in Caucasians (2). The incidence is higher among East Asians and cleft lip and palate patients and in the presence of cleidocranial dysplasia (3).

ST have been found in all areas of the dental arches, and may present in both the permanent and primary dentitions (4). They may be single, multiple, unilateral or bilateral in their distribution; nevertheless, there is a predilection for the premaxilla (5). Multiple ST are rare and mostly related to syndromes such as Gardner’s syndrome, cleidocranial dysplasia and cleft lip and palate. It has been reported that the prevalence rate for non-syndromic multiple ST is less than 1% (6,7).

Tooth eruption occurs as a continuous movement of tooth from its developmental position to the functional position (8). An impacted tooth is the one that fails to erupt into the dental arch within the expected time. The development of a supernumerary tooth with the crown in a deviated position such as an inverted tooth is less common (9,10). Inversion complicated by impaction is most frequently observed in the maxillary cuspid, followed by maxillary anterior ST, maxillary central incisors, lateral incisors and the mandibular third permanent molars (5,10). Complications related to an impacted ST include delayed eruption, retention of primary teeth, diastema, ectopic eruption, displacement or root resorption of adjacent teeth, most of which lead to occlusal problems (11). Retention and displacement of upper central incisors are the most frequent disturbance of eruption caused by unerupted ST in the upper jaw, between the incisors (12). Primordial or follicular cyst formation with significant bone destruction is also associated with ST. Nevertheless, ST particularly the inverted may present symptom-free anomalies that are coincidentally recognized in routine dental radiographic examination.

Literature covers research with large population samples about ST, however lacks series of inverted ST. Given that, the aim of this study was to investigate the clinical and radiographic manifestations and the related local complications in Turkish children with inverted ST.

## Subject and Methods 

This descriptive, retrospective study comprised patients with ST in inverted position, who were referred to the Istanbul University, Faculty of Dentistry, Department of Pediatric Dentistry and Department of Oral Surgery from January 2007 to May 2010. The inclusion criteria were to have: (I) radiographically detected supernumerary tooth, (II) ST in inverted position, (III) no specific clinical syndrome or systemic disease predisposing supernumerary teeth and (IV) no history of trauma or tumor in the oral cavity.

Orthopantomogram, periapical and occlusal radiographies of the anterior region were available for all patients. Some patients had additional 3D Computed Tomography (CT) images.

The study is in accordance with the ethical standards of the current revision of the Helsinki declaration of 1975. The patients’ parents were informed of the objectives of the study and informed consent was obtained from the patients’ parents or guardians.

The following data were supplied from patient records and documented: age, gender, medical and family history of the patients, status of dentition, number of ST, number of ST in inverted position, coexistence of ST in inverted and normal direction of eruption, location, orientation, morphology, clinical complications related to ST and management modality.

Location was identified as midline, right and left premaxilla. Regarding orientation in relation to permanent teeth, ST was recorded as normal and inverted. Morphologic features were defined as conical, incisiform and tuberculate. Clinical complications associated with ST are classified as diastema, local malocclusion, delayed eruption of permanent teeth, palatal swelling or follicular cysts. These judgments were made independently by two examiners (EBT, EK) and a consensus opinion was reached when a disagreement had occurred.

Statistical analysis of the data was performed using Number Cruncher Statistical System (NCSS) 2007& (Power Analysis and Sample Size) (PASS) 2008 Statistical Software (Utah, USA). The mean and standard deviation (SD) for each group was calculated. The findings were analyzed using Mann-Whitney U test to evaluate intergroup comparisons at a 5% level of significance. Qualitative data were compared by Fisher’s exact test was used.

## Results

A total of 38 patients with a mean age of 9.10±1.97 years (range: 6-13) and a strong male preponderance of 3.7:1 [30 males (78.9%); 8 females (21.1%)] were enrolled in the study. None of the patients suffered from syndromes known to predispose to ST such as cleft lip and palate or cleidocranial dysplasia and had a history of tumors or severe trauma to the oral cavity or head and neck region. No abnormalities in general growth and development were noted. Family history was unremarkable in regards of hyperdontia.

**Figure 1:**
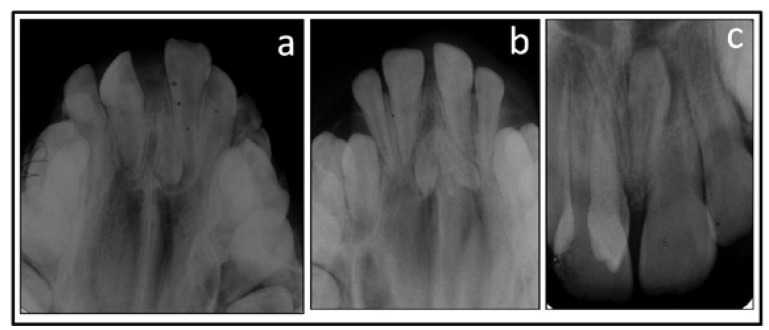
Occlusal radiograph showed one (a) and two (b) inverted supernumerary teeth at the midline, one inverted and one normal position supernumerary tooth (c).

The 38 patients enrolled in the study had a total of 69 impacted supernumerary teeth. Of these, 21 patients had two (55.3%), 15 patients had one (39.5%), and one patient had three and one patient had nine ST (2.6%). Among the 69 supernumerary teeth, 41 were in inverted position. Thirty five patients had one (92.1%), whereas 3 patients had two inverted teeth per case (7.9%) (Fig. 1).

All the ST in inverted position was located in the maxilla. Midline was the most frequent location for the single inverted super-numerary tooth in 18 (47.4%) patients, followed equally by the right and left premaxillary region in 10 patients each (26.3%). Paired inverted supernumeraries in three patients were located at both sides of midline.

Regarding morphology, 30 patients had conical (78.9%) and 8 (21.1%) had incisiform ST ( Table 1). Female patients presented only conical-shaped inverted ST, where males showed conical teeth (73.3%) and incisiform teeth (26.7%). No tuberculate-shaped ST was detected. Based on these data, gender has no statistically significant impact on tooth morphology (p>0.05).

**Table 1:**
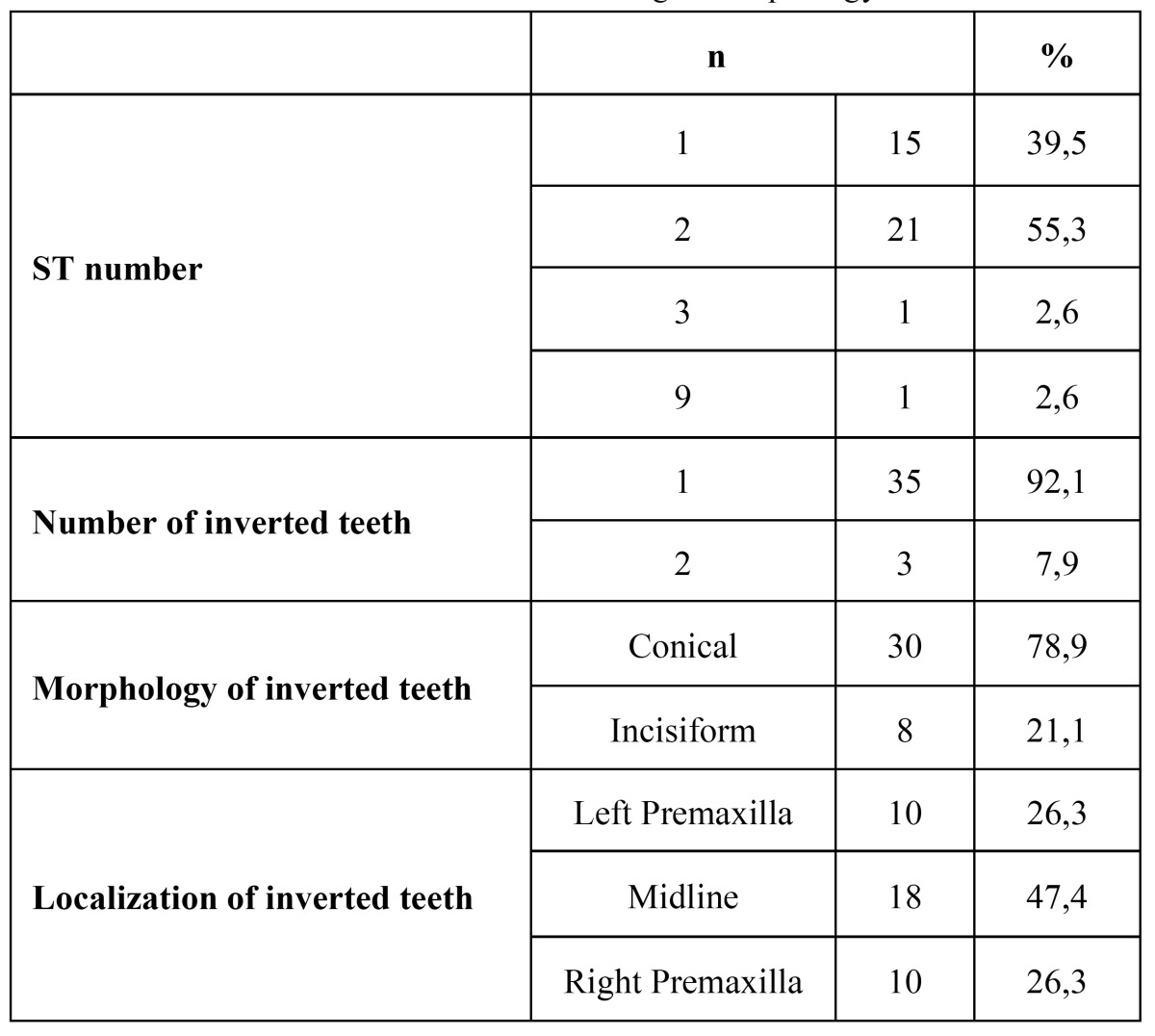
Distribution of inverted teeth according to morphology and localization.

**Figure 2:**
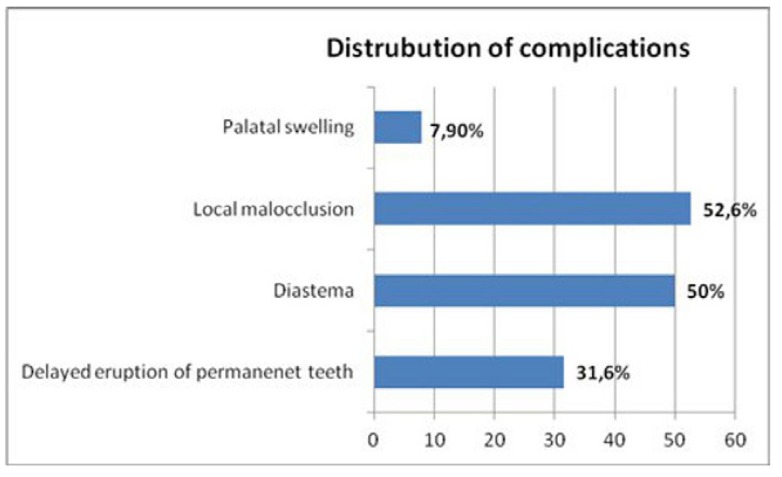
Clinical complications observed in patients.

Clinical complications associated with inverted ST were detected as diastema (50%), local malocclusion (52.6%), delayed erup-tion of permanent teeth (31.6%) and palatal swelling (7.9%), respectively (Fig. 2). There was no statistically significant difference between the number of inverted teeth and delayed tooth eruption, diastema, local malocclusion, palatinal swelling (p> 0.05). However, local malocclusion appeared as the most frequent complication related with single inverted ST (57.1%) ( Table 2).  Table 3 shows that there was no statistically significant difference between complications and age (p> 0.05).

**Table 2:**
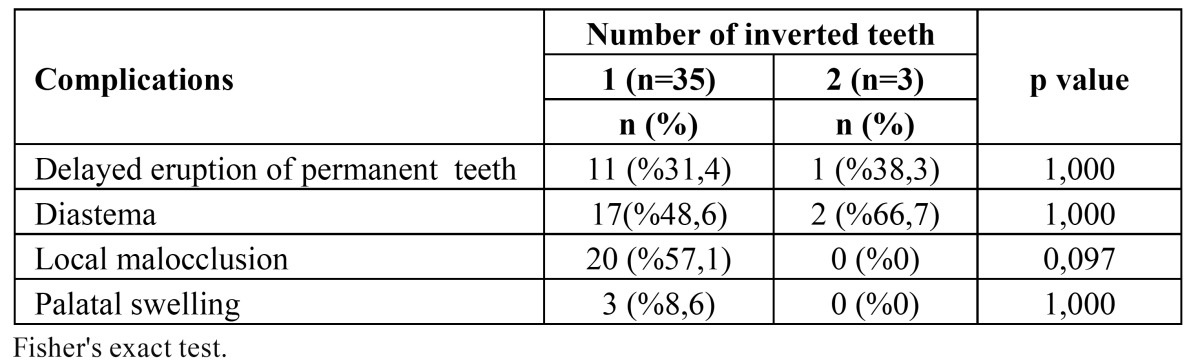
Frequency of complication acccording to inverted teeth number.

**Table 3:**
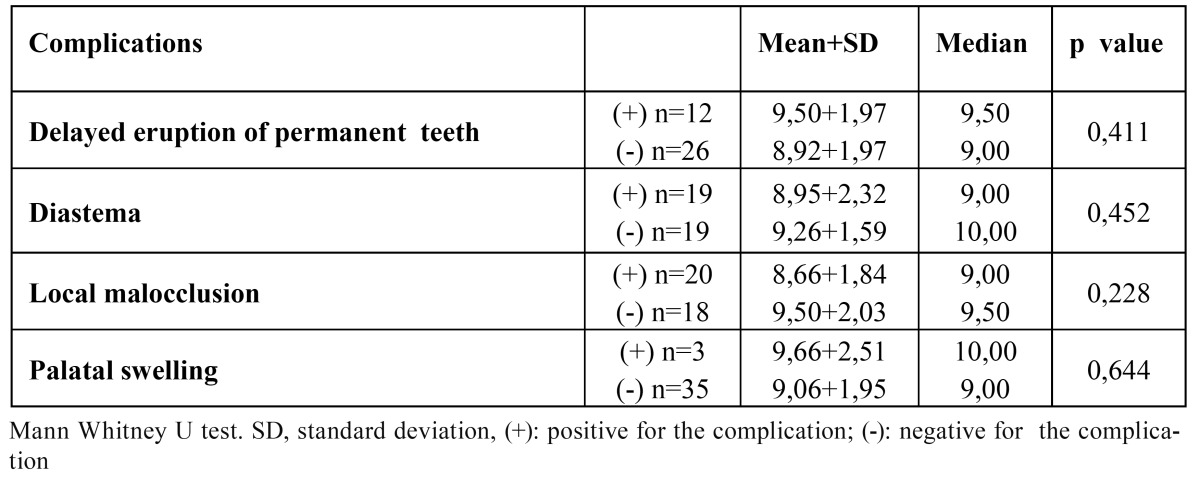
Evaluation of complications according to age.

Management of all the 38 patients comprised surgical intervention. Surgical intervention with subsequent follow-up during completion of permanent dentition was the main treatment approach in all cases. During this period, cases that required orthodontic treatment were directed to specialists for further management.

## Discussion

The present article demonstrates the demographic profile, and clinical and radiological features of Turkish children with ST, aiming to focus basically on the inversely oriented. Impacted ST are of great concern in children and young adolescents also to both dentists and the parents because of the existing complications or potential risks to proper function and desired esthetics and even of the related psychological effects. The inversion of ST more likely causes a tendency for such conditions (9,10). They are occasionally encountered in the clinical practices of pediatric dentistry and oral surgery (6) and clinical complications associated with ST are one of the major reasons why patients seek dental care (11).

Tooth impaction

The literature has reported a higher prevalence of ST in males (11,13). In a recent study conducted in a university hospital in Turkey, the prevalence of ST was found to be 1.2% among 3491 patients with a male predominance (Male/Female:1.8/1) (14). In our study it was observed that inverted ST are more frequent in males than females accordingly with the current literature. This strong male preponderance is similar with the prevalence reports of general Caucasian population, which varies from 1.3:1 to 2.5:1 (15).

Regarding the location of the ST within dental arch, most ST are reported to be localized between the maxillary central incisors (11,16). Our findings are in accordance with this observation and all the cases were located in the anterior maxillary arch. ST may be classified according to their morphology and location (13). The morphological classification includes conical, tuberculate, supplemental, and odontoma types. However, impacted inverted ST in conical shape are more common than those with incisiform shape (6,11). Consistent with previous studies, the findings of the present study revealed that most inverted and impacted ST were conical, and occurred in the premaxillary region.

Etiological factors

Odontogenic anomalies are the formative defects caused by genetic disturbances or environmental factors during tooth morpho-genesis (17). The favored hypothesis is that excessive growth of the dental lamina, or hyperactivity of the dental lamina has been cited as being responsible for the formation of additional tooth germs (18). According to this theory, the lingual extension of an additional tooth bud leads to a eumorphic tooth, while the rudimentary form arises from proliferation of epithelial remnants of the dental lamina induced by the pressure of the complete dentition (3). However, combinations of genetic and environmental factors were shown to be responsible factors for the occurrence of ST. Furthermore, with the available data indicating a strong hereditary component that does not follow a simple Mendelian pattern, some authors support a multifactorial mode of inheritance (5,6,19) and numerous case reports support a familial tendency.

Although several theories have been suggested about the etiology of ST, none has been proved. However, regardless of the tooth being a supernumerary or not, the direction of eruption is suggested to be influenced by either genetic or environmental factors, which are.

I. When associated with an inverted position of the tooth germ, the tooth may further that develop and erupt in an inverted position.

II. Some external force, such as tumors or traumatic extraction of deciduous teeth, inverts the tooth bud or the developing tooth.

III. It could be inversion by contact between the apex of an erupting tooth and the crown of an impacted supernumerary tooth (9,10).

Diagnostic approach

Detection of impacted ST is achieved using a thorough clinical examination and radiographic surveys. Comprehensive radiographic screening is required to establish an accurate diagnosis of ST and optimal management. In most cases of tooth impaction, a combination of radiographic views is needed to provide adequate information (9). Recently, 3D-CT images seem to be a good tool for the evaluation of an impacted tooth in the oral cavity. Since impacted tooth is always located at a higher position than the occlusal plane, dental radiographs may fail to reveal the whole image of an impacted tooth. However, CT may enable to make an accurate diagnosis of tooth impaction (20).

As in our case series, diagnosis and treatment planning were reached through a detailed clinical assessment. Radiological survey including orthopantomograph, periapical and occlusal radiographs and additionally 3D-CT views when necessary. We strongly suggest obtaining an orthopantomograph in order to detect any possible impacted ST, particularly in children at mixed dentition unless the patient had already one.

Complications

ST may erupt or remain unerupted. Due to their shape and volume they often hinder eruption and development of the permanent tooth related to them, causing crowding, displacement, diastema, retention, radicular resorption (21). It has been reported that in many cases, an inverted tooth was in direct contact with the cystic cavity and related teeth. Shaskiran et al. diagnosed an infected dentigerous cyst associated with an inverted central incisor. They enucleated the cyst successfully and extracted affected teeth. They concluded that the cyst resulted in the inversion of the permanent incisor due to pressure from cystic fluid, and also displaced the other affected teeth (22). If the ST are related to local disorders (diastema, delayed eruption or displacement) or if there is associated pathology, they should be extracted whenever the problem would be detected (15).

Management approach

The optimum time for the surgical removal of unerupted ST in many reports is hypothetical. There is no clear consensus as to what should be considered the best time period for surgical removal of unerupted ST, particularly in the premaxillary region (15). Given the ideal time for surgical intervention, “the earlier the offending supernumerary tooth is removed, the better the diagnosis” is the most relevant approach (9,23,24). Actually the age at the time of ST removal depends on the age of diagnosis. Early removal is recommended, particularly if it impedes the eruption of adjacent permanent teeth (24). Alternatively, such an inverted tooth could be extracted in early stage of development and autoreplanted with its follicle in the upright position (25). Nevertheless, treatment planning and timing should be analyzed individually for each case. Ideally, ST should be removed surgically whenever clinical or radiographic complications are found, a consensus in the literature (4,6,11,26). Inverted impacted teeth might require more complex surgery since they are apically positioned. In addition, increased vertical height with growth may make such teeth surgically less accessible. Therefore, early intervention is the preferred method in most cases (5). The developmental stage of the apex of adjacent permanent teeth should be considered prior to surgical management (11). In this study, management of inverted ST was decided depending on their locations, morphology and presence of complications. All cases in this study were subject to surgical removal to avoid the current complications becoming more severe and/or to prevent other future complications. The mean age for diagnosis and immediate removal of inverted ST in the present study group was nine, which is the age for completion of root formation in anterior incisors. Periodic recall visits are strongly advised to monitor the developing dentition as in our cases in order to avoid any damage to the adjacent tooth, cyst formation or other pathologic conditions.

This study highlights the demographic profile of inverted impacted ST and our lack of understanding of the reason of why the delayed eruption of the permanent tooth and local malocclusion. Further studies of ST concerning the inverted teeth particularly will be of value to determine how it could be possible to avoid complications and achieve successful management.

## Conclusion

In case of a long period of impaction, inverted ST may result in some complications. Early diagnosis and treatment of children with supernumeraries are important to prevent or minimize such kind of complications. Detailed clinical examination followed by a comprehensive radiographic screening is required to achieve an accurate diagnosis of inverted impacted teeth and optimal management. Periodic follow-up with radiographic examination is of great importance in childhood to detect any malocclusion in permanent dentition.
